# Supplementary material for: Iterative improvement in the automatic modular design of robot swarms
Source: PeerJ Comput Sci. 2020 Dec 7;6:e322. doi: 10.7717/peerj-cs.322 (PMC7924708; doi:10.7717/peerj-cs.322)
Supplement: Supplemental Information 3 [file peerj-cs-06-322-s003.zip › argos3/doc/api/standalone/a00528_source.html]

ARGoS: plugins/robots/generic/simulator/ground\_rotzonly\_sensor.cpp Source File


- Main Page
- Related Pages
- Namespaces
- Classes
- Files

- File List
- File Members

# plugins/robots/generic/simulator/ground\_rotzonly\_sensor.cpp

Go to the documentation of this file.

```
00001 
00007 #include <argos3/core/simulator/simulator.h>
00008 #include <argos3/core/simulator/entity/composable_entity.h>
00009 #include <argos3/core/simulator/entity/embodied_entity.h>
00010 #include <argos3/core/simulator/entity/floor_entity.h>
00011 #include <argos3/plugins/simulator/entities/ground_sensor_equipped_entity.h>
00012 
00013 #include "ground_rotzonly_sensor.h"
00014 
00015 namespace argos {
00016 
00017    /****************************************/
00018    /****************************************/
00019 
00020    static CRange<Real> UNIT(0.0f, 1.0f);
00021 
00022    /****************************************/
00023    /****************************************/
00024 
00025    CGroundRotZOnlySensor::CGroundRotZOnlySensor() :
00026       m_pcEmbodiedEntity(NULL),
00027       m_pcFloorEntity(NULL),
00028       m_pcGroundSensorEntity(NULL),
00029       m_pcRNG(NULL),
00030       m_bAddNoise(false),
00031       m_cSpace(CSimulator::GetInstance().GetSpace()) {}
00032 
00033    /****************************************/
00034    /****************************************/
00035 
00036    void CGroundRotZOnlySensor::SetRobot(CComposableEntity& c_entity) {
00037       m_pcEmbodiedEntity = &(c_entity.GetComponent<CEmbodiedEntity>("body"));
00038       m_pcGroundSensorEntity = &(c_entity.GetComponent<CGroundSensorEquippedEntity>("ground_sensors"));
00039       m_pcGroundSensorEntity->Enable();
00040       m_pcFloorEntity = &m_cSpace.GetFloorEntity();
00041    }
00042 
00043    /****************************************/
00044    /****************************************/
00045 
00046    void CGroundRotZOnlySensor::Init(TConfigurationNode& t_tree) {
00047       try {
00048          CCI_GroundSensor::Init(t_tree);
00049          /* Parse noise level */
00050          Real fNoiseLevel = 0.0f;
00051          GetNodeAttributeOrDefault(t_tree, "noise_level", fNoiseLevel, fNoiseLevel);
00052          if(fNoiseLevel < 0.0f) {
00053             THROW_ARGOSEXCEPTION("Can't specify a negative value for the noise level of the ground sensor");
00054          }
00055          else if(fNoiseLevel > 0.0f) {
00056             m_bAddNoise = true;
00057             m_cNoiseRange.Set(-fNoiseLevel, fNoiseLevel);
00058             m_pcRNG = CRandom::CreateRNG("argos");
00059          }
00060          m_tReadings.resize(m_pcGroundSensorEntity->GetNumSensors());
00061       }
00062       catch(CARGoSException& ex) {
00063          THROW_ARGOSEXCEPTION_NESTED("Initialization error in rotzonly ground sensor", ex);
00064       }
00065    }
00066 
00067    /****************************************/
00068    /****************************************/
00069    
00070    void CGroundRotZOnlySensor::Update() {
00071       /*
00072        * We make the assumption that the robot is rotated only wrt to Z
00073        */
00074       CRadians cRotZ, cRotY, cRotX;
00075       /* Set robot center */
00076       CVector2 cCenterPos;
00077       /* Position of sensor on the ground after rototranslation */
00078       CVector2 cSensorPos;
00079       /* Go through the sensors */
00080       for(UInt32 i = 0; i < m_tReadings.size(); ++i) {
00081          CGroundSensorEquippedEntity::SSensor& sSens = m_pcGroundSensorEntity->GetSensor(i);
00082          /* Get anchor position and orientation */
00083          cCenterPos.Set(sSens.Anchor.Position.GetX(),
00084                         sSens.Anchor.Position.GetY());
00085          sSens.Anchor.Orientation.ToEulerAngles(cRotZ, cRotY, cRotX);
00086          /* Calculate sensor position on the ground */
00087          cSensorPos = sSens.Offset;
00088          cSensorPos.Rotate(cRotZ);
00089          cSensorPos += cCenterPos;
00090          /* Get the color */
00091          const CColor& cColor = m_pcFloorEntity->GetColorAtPoint(cSensorPos.GetX(),
00092                                                                  cSensorPos.GetY());
00093          /* Set the reading */
00094          m_tReadings[i] = cColor.ToGrayScale() / 255.0f;
00095          /* Apply noise to the sensor */
00096          if(m_bAddNoise) {
00097             m_tReadings[i] += m_pcRNG->Uniform(m_cNoiseRange);
00098          }
00099          /* Is it a BW sensor? */
00100          if(sSens.Type == CGroundSensorEquippedEntity::TYPE_BLACK_WHITE) {
00101             /* Yes, set 0 or 1 */
00102             m_tReadings[i] = m_tReadings[i] < 0.5f ? 0.0f : 1.0f;
00103          }
00104          else {
00105             /* No, clamp the reading between 0 and 1 */
00106             UNIT.TruncValue(m_tReadings[i]);
00107          }
00108       }
00109    }
00110 
00111    /****************************************/
00112    /****************************************/
00113 
00114    void CGroundRotZOnlySensor::Reset() {
00115       for(UInt32 i = 0; i < GetReadings().size(); ++i) {
00116          m_tReadings[i] = 0.0f;
00117       }
00118    }
00119 
00120    /****************************************/
00121    /****************************************/
00122 
00123    REGISTER_SENSOR(CGroundRotZOnlySensor,
00124                    "ground", "rot_z_only",
00125                    "Carlo Pinciroli [ilpincy@gmail.com]",
00126                    "1.0",
00127                    "A generic ground sensor (optimized for 2D).",
00128                    "This sensor accesses a set of ground sensors. The sensors all return a value\n"
00129                    "between 0 and 1, where 0 means black and 1 means white. Depending on the type\n"
00130                    "of ground sensor, readings can either take 0 or 1 as value (bw sensors) or a\n"
00131                    "value in between (grayscale sensors). In controllers, you must include the\n"
00132                    "ci_ground_sensor.h header.\n\n"
00133                    "REQUIRED XML CONFIGURATION\n\n"
00134                    "  <controllers>\n"
00135                    "    ...\n"
00136                    "    <my_controller ...>\n"
00137                    "      ...\n"
00138                    "      <sensors>\n"
00139                    "        ...\n"
00140                    "        <ground implementation=\"rot_z_only\" />\n"
00141                    "        ...\n"
00142                    "      </sensors>\n"
00143                    "      ...\n"
00144                    "    </my_controller>\n"
00145                    "    ...\n"
00146                    "  </controllers>\n\n"
00147                    "OPTIONAL XML CONFIGURATION\n\n"
00148                    "It is possible to add uniform noise to the sensors, thus matching the\n"
00149                    "characteristics of a real robot better. This can be done with the attribute\n"
00150                    "\"noise_level\", whose allowed range is in [-1,1] and is added to the calculated\n"
00151                    "reading. The final sensor reading is always normalized in the [0-1] range.\n\n"
00152                    "  <controllers>\n"
00153                    "    ...\n"
00154                    "    <my_controller ...>\n"
00155                    "      ...\n"
00156                    "      <sensors>\n"
00157                    "        ...\n"
00158                    "        <ground implementation=\"rot_z_only\"\n"
00159                    "                noise_level=\"0.1\" />\n"
00160                    "        ...\n"
00161                    "      </sensors>\n"
00162                    "      ...\n"
00163                    "    </my_controller>\n"
00164                    "    ...\n"
00165                    "  </controllers>\n\n"
00166                    "OPTIONAL XML CONFIGURATION\n\n"
00167                    "None.\n",
00168                    "Usable"
00169                   );
00170 
00171 }
```

---

Generated on 10 Jul 2018 for ARGoS by 
 1.6.1
